# Determining the spatial heterogeneity underlying racial and ethnic differences in timely mammography screening

**DOI:** 10.1186/s12942-016-0067-3

**Published:** 2016-11-08

**Authors:** Joseph Gibbons, Melody K. Schiaffino

**Affiliations:** 1 Department of Sociology Health, San Diego State University, 5500 Campanile Dr., San Diego, CA 92182-4493 USA; 2 Graduate School of Public Health, San Diego State University, 5500 Campanile Dr., San Diego, CA 92182-4493 USA

**Keywords:** Timely mammograms, Geographically weighted regression, Spatial heterogeneity, Race/ethnicity, Community connection

## Abstract

**Background:**

The leading cause of cancer death for women worldwide continues to be breast cancer. Early detection through timely mammography has been recognized to increase the probability of survival. While mammography rates have risen for many women in recent years, disparities in screening along racial/ethnic lines persist across nations. In this paper, we argue that the role of local context, as identified through spatial heterogeneity, is an unexplored dynamic which explains some of the gaps in mammography utilization by race/ethnicity.

**Methods:**

We apply geographically weighted regression methods to responses from the 2008 Public Health Corporations’ Southeastern Household Health Survey, to examine the spatial heterogeneity in mammograms in the Philadelphia metropolitan area.

**Results:**

We find first aspatially that minority identity, in fact, increases the odds of a timely mammogram: 74% for non-Hispanic Blacks and 80% for Hispanic/Latinas. However, the geographically weighted regression confirms the relation of race/ethnicity to mammograms varies by space. Notably, the coefficients for Hispanic/Latinas are only significant in portions of the region. In other words, the increased odds of a timely mammography we found are not constant spatially. Other key variables that are known to influence timely screening, such as the source of healthcare and social capital, measured as community connection, also vary by space.

**Conclusions:**

These results have ramifications globally, demonstrating that the influence of individual characteristics which motivate, or inhibit, cancer screening may not be constant across space. This inconsistency calls for healthcare practitioners and outreach services to be mindful of the local context in their planning and resource allocation efforts.

## Background

Breast cancer persists as a leading cause of cancer death in women worldwide [1]. Early detection of breast cancer, defined as timely or guideline-concordant screening mammography and diagnosis contribute to survivorship. Specifically, stage 0–1 detection results in nearly 100% 5-year survival while stage IV detection only has a 22% survival rate according to a recent American Cancer Society estimate [2, 3]. Screening rates have risen among women, in particular for women in countries where screening was not previously available [3, 4]. However, disparities along the continuum of breast cancer persist among underserved women, a situation that reflects the experiences of underserved women everywhere. In particular, Non-Hispanic Black (henceforth, Black) women in the U.S. experience later stage diagnosis at much higher rates compared to White women [3, 4]. In addition, Hispanic/Latina women continue to experience lower comparable rates of timely screening mammography than both White and Black women as well as late-stage diagnoses comparable to Black women [4]. Recently, screening recommendations have also experienced variation with technological advances in screening modalities, changes in recommended ages for screening initiation contingent on genetics, familial history, and other nuanced risk factors have lead to the flattening of disparities [2, 5]. However, largely overlooked in this discussion is the role of spatial variation, or heterogeneity, in local screening rates. We argue that the spatial heterogeneity of mammograms by race/ethnicity helps to understand the disparities in rates overall, underlining the subtle role of local context on cancer screening.

While differences in outcomes across socially and racially/ethnically diverse populations are known, the role of local variation in breast cancer screening behaviors among underserved minority populations is not as well understood. Studies of geographic access to mammograms demonstrate disparities in screening rates by race/ethnicity, but often stop short of examining other contextual influences [6–9]. More subtle social, cultural, and other local factors are also found to shape timely cancer screenings [10–13]. We highlight for this study one’s community connection, group membership, and perceived medical discrimination as these factors are associated with healthy minority behaviors and vary at a local level [14–17], thus contributing to the risk of disease for minorities in a community [18, 19].

Community connection has been framed through a number of different terms, including social capital [14, 20] and collective efficacy [17]. It is derived from several measures including interpersonal trust with neighbors, a feeling of belongingness to the place, and the sense that residents share mutual interests [21]. Strong community connection within a group may facilitate leverage for treatment and survival by promoting timely screening. The protective effects of local ties can assist the spread of local health information such as where health services can be accessed, securing assistance in transportation to services, or the encouragement from peers to seek them out [17, 22, 23]. Membership in local community groups, ranging from churches to local nonprofits, provides another avenue to encourage service usage as it often puts members in contact with others outside of their proximate friend and family circle [23–25]. Group membership can be a facilitator to mobilize individuals toward healthy behaviors effectively [26, 27]. Put simply, the ability of friends or one’s pastor to inform and encourage one to seek out services like mammograms is more viable when these exchanges take place in a local day to day setting, such as a neighborhood.

The impact of community connection and group membership on health outcomes is noted to vary between racial/ethnic groups [14, 20, 26]. For example, community connection has been found to have a stronger positive effect on health outcomes among Latino populations, *ceteris paribus*, compared to the health outcomes of Black populations [28]. Sampson shows, in his study of collective efficacy, that the strength of community connection and group membership is not equal across space, being deeply stratified by local disparities such as racial segregation [17]. How these matter locally for mammograms for minority women is unclear. Dean and colleagues found that while local social capital influenced the relationship of Black women to mammography utilization, postulating a relationship with collective efficacy, they could not establish the direction of the relationship [14].

Another factor which may influence the use of health services by underserved minorities related to local context is discrimination from medical practitioners or medical discrimination. Medical discrimination as a barrier to health outcomes was widely described in the IOM report *Unequal Treatment* when it was one of the first empirical reports on the validated effect of medical discrimination on health outcomes [29]. Evidence regarding continued medical discrimination in health services experienced by women suggests this is a persistent issue that remains unaddressed [30, 31]. Jacobs et al. [32] found that medical discrimination related inversely to receiving screening mammography, they also found that more Black women compared to other groups reported health services discrimination.

While medical discrimination is a form of institutional racial discrimination, thus taking place within the larger context of the health service system, there is evidence that the perception of this discrimination for minority patients is not consistent across space. Studies on businesses and nonprofits, for example, have both found the institutional environment of professional settings is subject to local context [33, 34]. What’s more, Hunt et al. [15] found through a health survey on minority women that the perception of discrimination was lower in segregated communities. This evidence suggests medical discrimination may not be homogenous across communities and may be subject to spatial heterogeneity that warrants further study.

Examining the influence of local context on timely mammography requires an estimation strategy which accounts for granular variations of effects within a place. To this end, geographically weighted regression (GWR) is a novel way to examine the spatial heterogeneity in rates of timely mammography by race/ethnicity. Past studies have shown that GWR is an effective way to not only document local variations in health outcomes, [35] but also service usage [36]. While multi-level modeling strategies, commonly used in urban health research [37–40], can examine the interrelation between individual and neighborhood characteristics, they are limited in that they treat local effects as stationary and mutually independent across neighborhoods [41]. Multi-level strategies overlook the underlying spatial structures that would influence timely mammography rates within and between neighborhood boundaries.

To our knowledge, this is the first study to use GWR to understand the role of spatial heterogeneity to explore within race and social category variation in the utilization of timely screening mammography. The expected contributions of these findings relate to the potential of GWR as a tool for healthcare professionals better understand nuance *within* places to improve patient- and community-centered responses to the need for timely mammography that may not always be easily answered by broader designations. Further, our results suggest other factors such as social and spatial determinants also need to be considered or re-configured. The objectives of this analysis are to assess the spatial factors associated with timely mammography utilization in a cohort of women. With GWR, we can compare the local variation in our predictors localized population parameters at the census tract level. Through this comparison, we can begin to contextualize the spatial relationship of population factors to timely mammographies among women in the study sample, isolating potential neighborhood impacts on the local spatial structure.

## Timely mammography theoretical and conceptual foundations

**Fig. 1:**
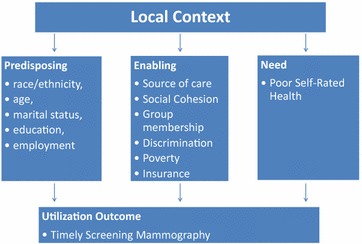
Revised Andersen behavioral model

Variation in the utilization of timely mammography outcomes is multi-dimensional and complex. The *Andersen Model of Health Services Utilization* is a valuable model that takes into account this complexity and offers a framework that allows us to adapt, conceptualize, and study these dimensions for our present analysis [42]. Broadly, Andersen describes multi-level factors that operationalize the complexity of access to care and utilization as a product of how multiple social, contextual, and perceived factors can influence our utilization or lack thereof. As Fig. 1 shows, these factors are operationalized as *predisposing*, or background characteristics which shape a person’s inclination to seek out healthcare and are not mutable. For example, African–Americans are less likely to find care due to historical systemic racism within the health system [43, 44]. Next, *enabling* factors include those which facilitate or hinder, if absent, one’s efforts to find healthcare. For example, lacking insurance makes it nearly impossible for one to obtain timely and affordable healthcare. Finally, *need* factors reflect ailments a person might be experiencing that would require healthcare in the first place, these are subject to a perceived and evaluated need that can be influenced by discrimination when they do visit a doctor. The Andersen Model has been adapted successfully in multiple cases, and part of the strength of this model is its adaptability to study context and outcomes related to health utilization behavior [45]. With the present analysis, we adapted the Andersen Model to include *local* context as it relates to spatial heterogeneity across all levels of need from predisposing to outcome.

## Hypotheses

To address the potential effect of local context on cancer screening disparities, our study explores the spatial heterogeneity in factors associated with timely mammography within racial and ethnic minority populations. To this end, we propose the following hypotheses:

1. Utilization of timely screening mammography by Latina/Hispanic women will be negatively associated versus non-Hispanic women, and will not vary significantly between Black women versus White women.
  - Utilization of timely mammography will vary significantly by geography among Black women.
  - Utilization of timely mammography will vary significantly by geography among Hispanic women.
2. Utilization of timely screening mammography will be positively associated with community connection.
  - Utilization of timely screening mammography will vary significantly by geography among respondents reporting community connection.

## Data and methods

### Data source

To empirically examine our hypotheses, we used a sample of female respondents from the 2008 Public Health Management Corporation (PHMC) Southeastern Pennsylvania Household Survey (N = 3261) with geocodes to link to the 2005–2009 American Community Survey (ACS) geographic dataset consisting of approximately N = 998 census tracts. The goal of the PHMC is to collect the information on individual’s health status, behaviors, attitudes, and access to healthcare in the following five counties of the Philadelphia metropolitan area [46]. PHMC respondents used in this study are those eligible to receive guideline concordant recommendations appropriate for the data collection time period of 2008, specifically women aged 40 and over [47]. While the U.S. Preventive Services Task Force has since suggested a reduced marginal benefit in the range of participants to include in population-based screening mammography [48], for the purposes of the present analysis we included the population appropriate to the time period. On the reliability and validity of the PHMC surveys, a recent study [21] reported that several health and socioeconomic indicators (e.g., obesity rate and poverty) drawn from PHMC data were comparable with those estimated by the Centers for Diseases Control and Prevention.

### Measurements

The dependent variable is the self-reported use of timely *Breast Cancer Screening Mammography*. Participants were asked if they had received a screening mammography within the guideline concordant time frame recommended by their medical practitioner for the time period in which data were collected. Following the common practice, we dichotomized the answers into no (coded 0, reference group) and yes (coded 1). Our predictors were determined based on Andersen’s Behavioral Model [42, 49], including Predisposing, Enabling and Need factors which would compel one to seek out medical services like a mammography. Starting with Predisposing factors, our focal predictors are *race/ethnicity*; the PHMC classified respondents into non-Hispanic White (reference group, hence just White), Black, Hispanic/Latinas, and non-Hispanic other minorities. Three race/ethnicity dummy variables were included in the analysis. Other predisposing covariates include age, poverty, race/ethnicity, marital status, employment status, and education attainment. Respondents reported their ages in years, and we treated *age* as a continuous variable. In keeping with the screening guidelines circa 200 [8, 48] we restrict our sample to age 40 and above. *Marital status* was categorized into four groups: single (reference group), married or living with a partner, widowed/divorced/separated (WDS), and another marital status. Gender was not included as a predictor given the surveys focus on female breast cancer screening.

Turning to Enabling factors, we add as focal variables community connection and medical discrimination given their strong association with race. First, we include a measure of *Medical discrimination*; the respondents were asked if they have ever experienced discrimination when getting medical care because of their race, ethnicity, or color. Those who perceived medical discrimination were coded 1, otherwise 0. Next, we include a measure of *community connection*, a composite score based on the principal components analysis (PCA) of respondents’ answers to the following three questions: (1) *Willingness*, “Would you say that most people in your neighborhood are always, often, sometimes, rarely, or never willing to help their neighbors?” From *always* to *never*, we coded from 5 to 1. (2) *Belonging*, “Do you strongly agree, agree, disagree, or strongly disagree that you feel that you belong and are part of your neighborhood?” We coded the answers with a four-level Likert-type scale where 4 means *strongly agree,* and 1 indicates *strongly disagree*. (3) *Trust*, “Do you strongly agree, agree, disagree, or strongly disagree with the statement that most people in your neighborhood can be trusted?” The coding scheme is also a four-level Likert-type scale (4 = *strongly agree*, and 1 = *strongly disagree*). The PCA results suggested that one factor is sufficient to capture over 60% of the variance among these three questions. We used the regression method to obtain the factor score as our dependent variable (with a mean of 0 and a standard deviation of 1). A higher score indicates stronger community connection.

Also, we include enabling factors more commonly found in behavioral models [49], consisting of those who lived under the federal poverty line as a measure of the financial situation, coding 1 as in *poverty* and 0 otherwise. For *employment status*, we classified those will full-time employment status as employed. Next, we include a measure of *insurance status*; a respondent coded 1 when a respondent reported that she had health insurance, otherwise 0. Next, we included variables for *Source of Health Care*, where an individual goes to get medical services, as a way to understand healthcare access. We categorized the answers into four groups: private doctor’s office, community health center or public clinic, outpatient clinic, and other places (e.g., hospital emergency room). To test our hypotheses, the “other places” category was used as the reference group, and three dummy variables were considered in the analysis. We also include a measure of *Local group participation,* the total number of local groups that a respondent participates in such as social, political, religious, school-related, and athletic groups. Finally, we include a measure of residence in the city and county of Philadelphia, *City*.

Finally, for factors of *Need*, we use a measure of *self*-*rated health*. The respondents were asked to evaluate their health as poor, fair, good, very good or excellent. Their answers were further dichotomized into poor/fair (coded 1) and good/very good/excellent (coded 0), which is a conventional practice. While it is common in GWR studies using administrative units like census tracts to utilize the geographic centroids of the unit as a proxy of the individual level, this approach has been criticized for underestimating the spatial variation across research area [41]. To address this issue, and following the precedent established by previous studies, we used ArcGIS to generate coordinates for each respondent that fall at random within their respective census tract [41, 50]. To ensure the reliability of this approach, multiple coordinates were generated for each observation and sensitivity analysis were conducted (results available on request). This approach of spatial randomization has been found to be a useful method to preserve spatial variation [41].

### Analytic methods and strategy

To explore the spatial variation between timely mammograms and other covariates across the Philadelphia metropolitan area, we employed logistic GWR to handle the binary dependent variable [51]. As we randomly created the coordinates for each individual, the model below can be applied to our data:

$$\log \left( {\frac{{y_{i} }}{{1 - y_{i} }}} \right) = \beta_{0i} \left( {u_{i} ,v_{i} } \right) + \mathop \sum \limits_{n = 1}^{k} \beta_{ni} \left( {u_{i} ,v_{i} } \right)*x_{ni}$$

where *y*_*i*_ is the probability of reporting timely mammograms for an individual *i*, (*u*_*i*_, *v*_*i*_) denotes the coordinates of individual *i*, *x*_*ni*_ represents the explanatory variables (n = 1, …, k) discussed above for individual *i*, and *β*_*ni*_ represents the estimated association of variable *n* with mammograms for individual *i*. We used the software program developed by Fotheringham et al. [51] to implement the analysis. The estimation method is the iteratively reweighted least squares and the kernel density function is the bi-square weighting function, which is a commonly used weighting scheme [51]. When the data points are dense in a study area, the choice of kernel density function may not affect the results greatly.

One advantage of GWR is that it is an extension of generalized regression models, and thus the interpretations of regression coefficients remain unchanged [52–54]. Explicitly, the regression coefficient of a specific variable at a specific location, (*u*_*i*_, *v*_*i*_), in the model above indicates the change in the log-odds of having a timely mammograms given a one-unit change in this variable. Similar to the conventional logistic regression, exponentiating the coefficient yields the odds ratio associated with this variable at a particular location. As the model above generates results for each individual in our data, it is ineffective to show all local estimates. Following previous studies [41, 53, 55], we reported the estimates of conventional logistic results, presented the five-number summary (i.e., minimum, three quartiles, and maximum) of local estimates, and visualized the GWR results with thematic maps using a recently developed method [50]. The corrected Akaike Information Criterion (AIC) was used to understand whether the logistic GWR fits the data better than the conventional logistic model [51]. As a rule of thumb, when the difference in AICs between two models is larger than 4, the model with the smaller AIC is strongly preferred [56].

## Results

### Aspatial results

Table 1 presents the descriptive statistics for this study. Overall, 74.03% of the PHMC respondents received timely screening mammographies. As for racial composition, the 2008 PHMC survey included 70.84% of White, 22.85% of Black, 3.93% of Hispanic/Latinas, and roughly 2.39% of non-Hispanic other minority groups. These figures closely matched to those reported by the 2005–2009 ACS. Most of those surveyed, 95.95%, had some insurance. Only 6.10% reported experiencing medical discrimination. As for healthcare access, most respondents went to a private practice for regular care, 88.87%, compared to a community health center, which amounted to only 5.24% of those surveyed. Regarding other individual characteristics, 6.69% of the interviewees did not complete high school, while more than 40.08% of the individuals had a college degree or greater. As for group membership, most respondents reported membership in at least one group. Community connection was not reported in Table 1 as it is a means-centered variable.

**Table 1:** Descriptive statistics

Outcome variable	Count^a^	(%)
Received timely screening		
Yes	2414	74.03
No (ref)	847	25.97
*Predisposing variables*		
Age (average)	56.68	–
Race/ethnicity		
White (ref)	2310	70.84
Black	745	22.85
Hispanic/Latina	128	3.93
Other race	78	2.39
Educational attainment		
No high school diploma (ref)	218	6.69
High school	1077	33.03
Some college	659	20.21
College or greater	1307	40.08
Marital status		
Married	1761	54.00
Not married (ref)	1500	46.00
*Enabling*		
Poverty status		
Lives below 100% FPL	260	7.97
Above 100% federal poverty level (ref)	3001	92.03
Fulltime employment status		
Yes	1918	58.82
No (ref)	1343	41.18
Insurance status		
Yes	3129	95.95
No (ref)	132	4.05
Source of healthcare		
Other center (ref)	65	1.99
Community health center	171	5.24
Private practice	2898	88.87
Outpatient clinic	127	3.89
Experienced medical discrimination		
Yes	199	6.10
No (ref)	3062	93.90
Respondent Residence		
Urban (Philadelphia Residence)	1377	42.23
Suburban (ref)	1884	57.77
Group participation (average)	1.224	–
*Need*		
Self-rated health status		
Poor or fair	732	22.45
Good, very good or excellent (ref)	2529	77.55
N	3261	

^a^Numbers are total counts unless otherwise noted

Table 2 presents the global, or conventional logistic findings. The results for predisposing factors are somewhat surprising, given the previous literature. Both Black and Hispanic/Latina women reported greater odds of getting timely screening mammograms. Being Black increases the odds of a timely mammogram by 74% (1.738 − 1 = 0.738; *p* < 0.01) while being Hispanic/Latina increases the odds by 80% (*p* < 0.0501). The other predisposing factors are more in line with the past literature. A college education (or greater) and being married both increase the likelihood one will get a mammogram. Turning to enabling factors, employment and having insurance both increase the odds one will get a timely mammogram. Also, where one goes for healthcare consistently has an important role in screening. Based on our findings, any place other than a center like a hospital will increase odds of a timely mammogram. Access to a community health center appears to matter the most in encouraging a mammogram. Meanwhile, experiencing medical discrimination was inversely related to reporting receipt of a timely mammogram though this association was not significant (AOR 0.784). What is more, community connection was not significant in the global models and membership in groups only had a marginally significant effect. Turning lastly to need, women with poor/fair self-rated health reported 30% lower odds of receiving a mammogram in a timely manner by 30% (*p* < 0.01).

**Table 2:** Global logistic regression results of breast cancer screening (1 = yes; 0 = no)

Variable	Odds ratio	95% confidence interval		Coefficient	S.E.	Significance
*Predisposing*						
Race/ethnicity (ref. = non-Hispanic White)						
Black	1.738	1.371	2.203	0.552	0.120	***
Hispanic/Latina	1.799	1.126	2.875	0.587	0.239	**
Other race	0.726	0.444	1.187	−0.319	0.250	
Age	1.000	0.992	1.009	0.001	0.004	
Educational attainment (ref. = no high school diploma)						
High school	1.092	0.781	1.529	0.088	0.171	
Some college	1.068	0.743	1.535	0.065	0.185	
College or greater	1.349	0.941	1.935	0.299	0.184	
Married	1.304	1.094	1.554	0.265	0.089	**
*Enabling*						
Poverty status (1 = poor, 0 = non-poor)	0.979	0.714	1.341	−0.021	0.160	
Employment status (1 = employed, others = 0)	1.206	0.994	1.463	0.186	0.098	*
Insured (1 = having health insurance, 0 = no health insurance)	3.857	2.616	5.687	1.349	0.198	***
Source of healthcare						
Community health center	2.728	1.449	5.138	1.003	0.322	**
Private practice	2.053	1.228	3.432	0.719	0.262	**
Outpatient clinic	1.671	0.882	3.166	0.513	0.326	
Experienced medical discrimination	0.784	0.563	1.092	−0.243	0.168	
Community connection	1.056	0.988	1.130	0.054	0.034	
Group membership	1.060	0.996	1.128	0.058	0.031	*
Respondent residence—city (ref = suburban)	1.026	0.849	1.240	0.025	0.096	
*Need*						
Poor or fair self-rated health	0.700	0.570	0.861	−0.356	0.105	***
Constant				−1.475	0.462	***
N	3261					
Akaike Inf. Crit.	3613.910					

* *p* < 0.1; ** *p* < 0.05; *** *p* < 0.01

### GWR results

**Fig. 2:**
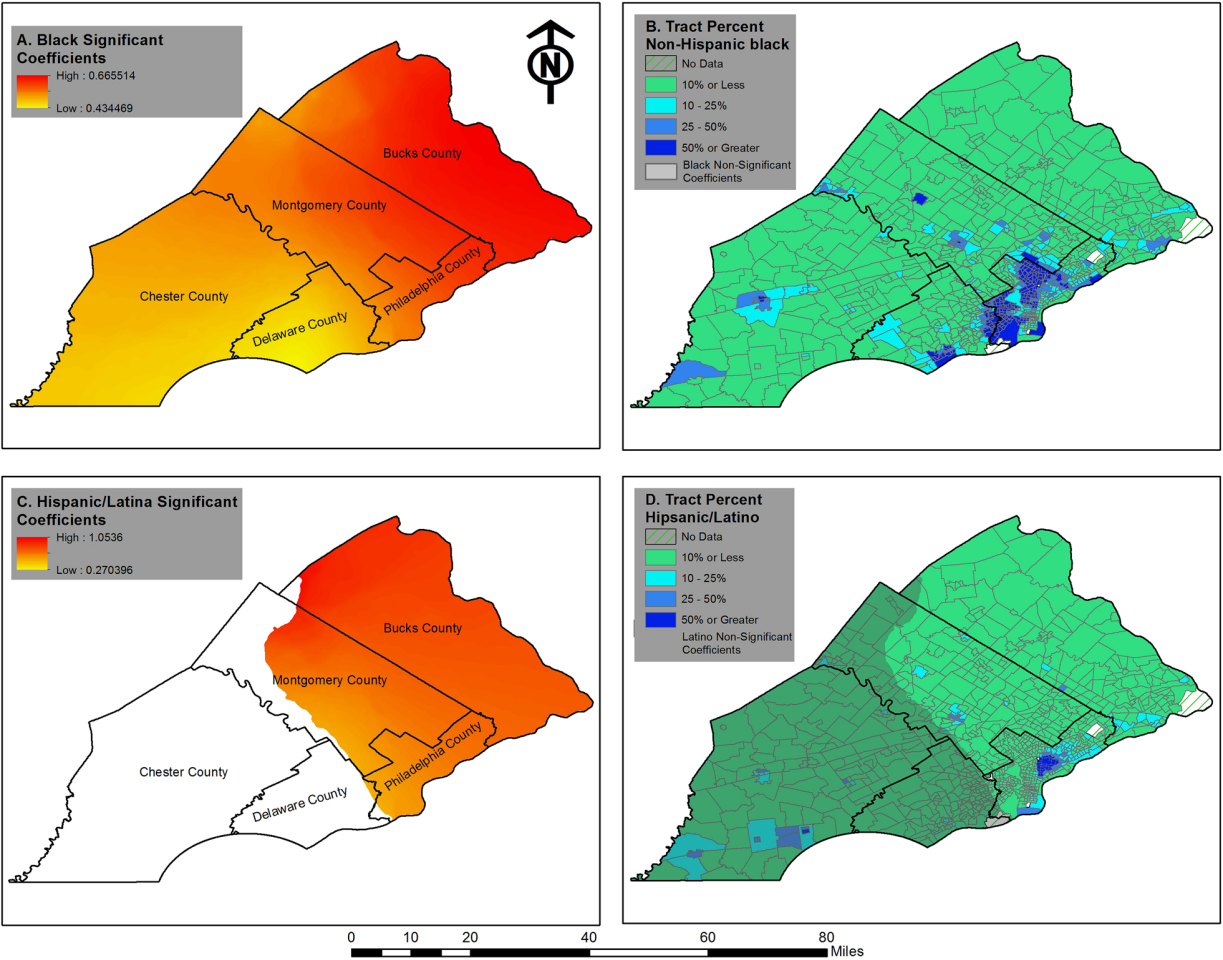
GWR of breast cancer screening and race in the Philadelphia metropolitan area

**Fig. 3:**
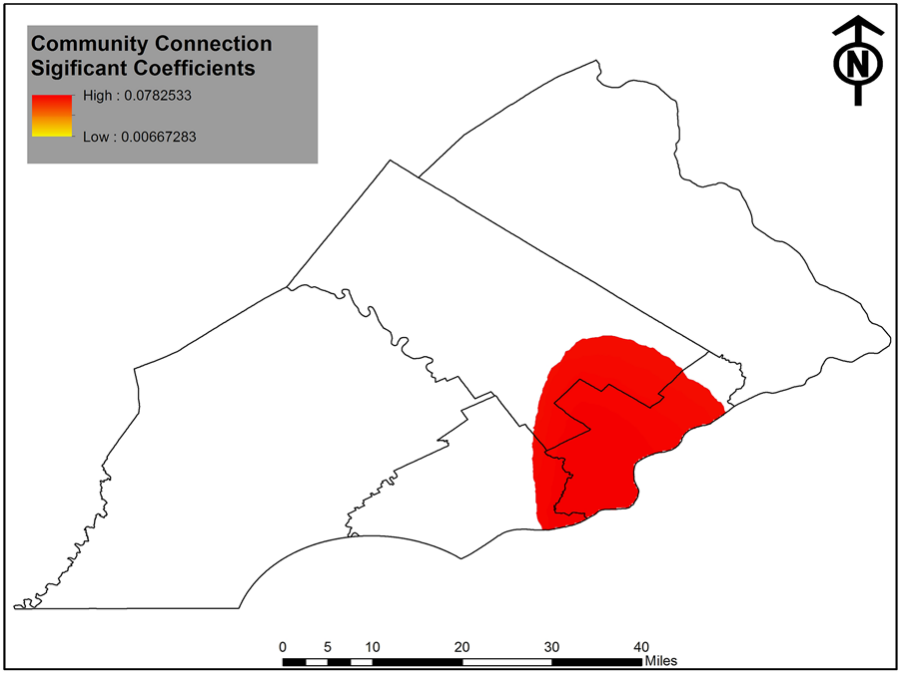
Breast cancer screening and community connection in Philadelphia metropolitan area

GWR logistic regression generated a set of coefficient estimates for each individual, which makes it difficult, if not impossible, to present all results. Following Fotheringham et al. [51], we reported the five-number summary in Table 3 and visualized the GWR findings into thematic maps. The goal of this table is to present the spatial range in magnitude of the variable coefficients. Local statistical significance for select GWR coefficients is mapped out in Figs. 2 and 3. While several methods have been proposed to examine spatial heterogeneity of significance and coefficients [57, 58], these methods are not applicable to the logistic GWR model and visualization remains an appropriate way to explore this spatial heterogeneity.

**Table 3:** Five-number summary of the GWR logistic regression results; bandwidth 3000

Variable	Min	Q1	Median	Q3	Max
*Predisposing*					
Race/ethnicity (ref. = non-Hispanic White)					
Black	0.434	0.516	0.561	0.602	0.666
Hispanic/Latina	0.270	0.485	0.601	0.683	1.054
Other race	−0.734	−0.368	−0.214	−0.148	−0.091
Age	−0.007	−0.002	0.001	0.003	0.004
Educational attainment (ref. = no high school diploma)					
High school	−0.261	−0.104	−0.065	−0.021	0.338
Some college	−0.170	−0.156	−0.145	−0.061	0.622
College or greater	−0.056	−0.019	0.012	0.070	0.818
Married	0.209	0.248	0.270	0.298	0.388
*Enabling*					
Poverty status (1 = poor, 0 = non-poor)	−0.214	−0.184	−0.172	−0.123	0.285
Employment status (1 = employed, others = 0)	0.063	0.131	0.191	0.214	0.241
Insured (1 = having health insurance, 0 = no health insurance)	1.147	1.438	1.476	1.502	1.755
Source of healthcare					
Community health center	0.818	0.846	0.857	0.900	1.352
Private practice	0.464	0.502	0.528	0.609	1.060
Outpatient clinic	0.289	0.302	0.348	0.505	1.031
Experienced medical discrimination	−0.494	−0.191	−0.144	−0.126	−0.106
Community connection	−0.004	0.065	0.076	0.079	0.080
Group membership	−0.033	0.036	0.062	0.075	0.099
Respondent residence—city (ref = suburban)	−0.022	0.015	0.041	0.053	0.092
*Need*					
Poor of fair self-rated health	−0.575	−0.404	−0.368	−0.353	−0.259
Intercept	−2.354	−1.457	−1.344	−1.174	−0.759
Akaike Inf. Crit.	3609.497				

*Min* minimum, *Q1* first quartile, *Q3* third quartile, *max* maximum

On the question of whether the GWR logistic model fit our data better than the global logistic model, we compared the corrected AICs in Tables 2 and 3. Because the GWR AIC is smaller than the global AIC by 4, it indicates the GWR provides superior fit for our predictors. As Table 3 shows, the GWR estimates range quite dramatically, suggesting that the relationships between our independent variables and receipt of timely mammograms may depend on where an individual resides. This offers support to the importance of geographically weighted results over the global results. Starting with our focal predisposing predictors, the maximum size of the coefficient for being Hispanic/Latina is nearly 4 times as large as its minimum, suggesting substantial variation in how being Hispanic/Latina impacts timely mammograms. The coefficients for Blacks also increase, albeit not as dramatically. These results mean that the impact of race on mammograms is not consistent across the region. Turning to our focal enabling variables, community connection, group membership, and medical discrimination also vary, although most notably there are some local coefficients for which community connection relates negatively to mammograms.

To better contextualize our GWR estimates, we make use of a series of maps of the region to unpack the local spatial relations for Black and Hispanic/Latina coefficients, presented in Fig. 2. To help with the easy interpretation, we first created the spatially smoothed local estimates and local t-values with the GWR results. We then overlaid local estimates with t-values in the geographic information systems and showed the local estimates with a t-value that is greater than 1.96 (*p* value <0.05). That is, the colored areas were estimated to have statistically significant associations of covariates with receipt of timely mammograms. We used the red–orange gradient scheme to show different magnitudes of the local estimates, red signifying strong effects and orange indicating weak associations. Second, in a separate set of maps we then overlaid the areas with insignificant coefficients (with t-values between −1.96 and 1.96) on top of census tract data displaying ACS estimates. While one should proceed with caution in interpreting these visuals without multi-level models, given the risk of ecological fallacy, they do provide some indication of the context as to why the significant coefficients are located where they are.

The localized coefficients for Hispanic/Latinas present an interesting find. These results show that the higher odds of receiving timely mammography among Hispanic/Latina is only significant in roughly half of the region, especially in the suburban Bucks County, not across all respondents in that ethnic category as regression results suggest in Table 1. This is unexpected for one as this area only has a few large Hispanic populations, suggesting that ‘being Hispanic/Latina’ matters for reasons other than being in a mostly Hispanic area. Spatial heterogeneity for Black coefficients, in contrast, are significant across the region, growing in strength as one moves east. The lowest coefficients are generally found in Delaware County. It is not clear, based on where the mostly Black populations are found, why this variation exists as all counties have areas with large Black populations, although Philadelphia has the strongest concentrations. One possible explanation why Delaware County has the lowest coefficients is that it is only of the region that does not directly share a border with Philadelphia or inner ring suburban communities, and thereby is the furthest from the largest Black populations.

Turning to our enabling variables of community connection and medical discrimination we find spatial findings of interest. First, Fig. 3 reveals the coefficients for community connection were significant in select parts of the region, encompassing most of the city of Philadelphia and its immediate surrounding areas. This is notable as community connection was not significant in the global model. Comparing this map to the ACS data in Fig. 2 shows that the significant coefficients appear to co-occur in the areas where the highest concentrations of Black and Hispanic populations are found. These results do not mean that no other area of the region lacked community connection, but our findings do suggest that there is a significant relationship between community connection and women seeking out mammograms that is confined spatially to the area presented in the figure.

## Conclusion

Broadly, our results report greater odds of timely screening mammography among racial and ethnic minority populations that appear to be better for this well-insured cohort study sample. However, our primary study purpose, the study of spatial heterogeneity, illustrates a salient point. Geographically weighted regression results support our hypotheses that spatial heterogeneity exists in timely mammograms among Black and Hispanic/Latina women as they compare to white women, and what appear to be greater odds of timely mammography among the whole racial/ethnic group may in fact be limited. In addition, we found that other predisposing and enabling factors like community connection also vary substantially over space. This presents an important innovation to our understanding of health service provision, demonstrating the overlooked role local context carries when considering Andersen’s Behavioral Model of utilization. While racial/ethnic groups are typically considered homogenous, our findings show that unaccounted variation across space and place exist within these groups, even when accounting for standard controls like socio-economic and demographic variables. This illustrates that social factors persist even among the insured as we saw that health status persisted as a barrier to timely care.

While GWR is an exploratory tool, comparing the GWR maps to one another, as well as to the neighborhood census tract data, reveals patterns allowing informed speculation as for the role race/ethnicity has on mammography. First, significant Hispanic/Latina coefficients are mainly found in the suburban counties of Bucks and Montgomery. One’s Hispanic/Latina identity thus appears to matter in encouraging mammographies in these suburban areas. This may reflect recent patterns of immigration in the United States as Hispanic migrants have increasingly dispersed into suburban and rural ‘new destinations’ as opposed to concentrating in cities [59]. Future research should investigate screening practices for suburban Hispanic/Latinas to understand this trend better. Second, while the coefficients for Black respondents are significant and positive across the region, a close analysis of the other GWR results suggests a more localized dynamic is taking place. Community connection’s effect in encouraging mammograms is localized to a mostly Black and Hispanic area. This could be a reflection of a phenomenon known as ‘ethnic density.’ Ethnic density a process identified in several countries wherein minorities residing in mostly minority communities, such as places racially segregated, gain protective health effects from the close connections and reduced discrimination enjoyed in these places [38, 60, 61]. Indeed, it would support Dean’s et al. [14] theory that Black women are more likely to pursue mammograms in their local context based on the presence of local community connection.

There are a number of possible considerations for the high overall mammography utilization rates for minority women, including income and insurance status. Indeed, insurance was one of the most salient predictors in our models, which is not surprising given our highly insured study population. On the other hand, high overall mammography utilization among minorities could be a reflection of high levels of community health centers in the city of Philadelphia. Indeed, our results show these centers had the strongest predictive power on mammograms. Laiteerapong et al. [62] suggested that Black women visit community health centers like Federally Qualified Health Centers (FQHCs) at rates greater than White or Hispanic/Latina women and that mammograms are also more likely to occur among FQHC attendees, suggesting a positive effect of FQHC utilization. These results could also be affected by the disproportionate representation in the PHMC of respondents with high levels of socio-economic status or some other unmolded factors unique to Philadelphia. Future research should seek to replicate this analysis in other regions to determine the singularity of our study. While the exact spatial character of race/ethnicity’s relation to screening is likely to vary based on location, the bottom line is that the impact of local context on mammography matters differently for racial/ethnic groups across space, a finding likely to be applicable globally.

Timely mammography screening is the first step in understanding and acting to mitigate the devastating impact of breast cancer. There is substantial literature supporting the need for better access to timely screening and care; we lack an understanding of the localized racial/ethnic, cultural and economic factors that continue to make these barriers persist. It is not sufficient to aspatially examine the predisposing and enabling factors that facilitate or bar access to timely screening mammograms among racial/ethnic minorities. Indeed, as our results show, the impact of one’s race/ethnicity on pursuing mammograms, as well as other intervening variables, changes from one area to another. Thus, efforts to ensure equitable screening rates among groups must investigate local potential variations in their instances, seeking to determine why these disparities exist and, when necessary, how to manage them.

## Abbreviations

GWR: geographically weighted regression
PHMC: Public Health Management Corporation
ACS: American Community Survey
AIC: Akaike Information Criterion
FQHC: Federally Qualified Health Centers
